# Patient Derived Xenografts for Genome-Driven Therapy of Osteosarcoma

**DOI:** 10.3390/cells10020416

**Published:** 2021-02-17

**Authors:** Lorena Landuzzi, Maria Cristina Manara, Pier-Luigi Lollini, Katia Scotlandi

**Affiliations:** 1 Laboratory of Experimental Oncology, IRCCS Istituto Ortopedico Rizzoli, 40136 Bologna, Italy; mariacristina.manara@ior.it; 2 CRS Development of Biomolecular Therapies, Laboratory of Experimental Oncology, IRCCS Istituto Ortopedico Rizzoli, 40136 Bologna, Italy; 3 Interdepartmental Center for Cancer Research (CIRC), University of Bologna, 40138 Bologna, Italy; pierluigi.lollini@unibo.it

**Keywords:** osteosarcoma, patient-derived xenograft (PDX), PDX-derived cell lines, mouse PDX clinical trial, genome-driven therapy, personalized therapy.

## Abstract

Osteosarcoma (OS) is a rare malignant primary tumor of mesenchymal origin affecting bone. It is characterized by a complex genotype, mainly due to the high frequency of chromothripsis, which leads to multiple somatic copy number alterations and structural rearrangements. Any effort to design genome-driven therapies must therefore consider such high inter- and intra-tumor heterogeneity. Therefore, many laboratories and international networks are developing and sharing OS patient-derived xenografts (OS PDX) to broaden the availability of models that reproduce OS complex clinical heterogeneity. OS PDXs, and new cell lines derived from PDXs, faithfully preserve tumor heterogeneity, genetic, and epigenetic features and are thus valuable tools for predicting drug responses. Here, we review recent achievements concerning OS PDXs, summarizing the methods used to obtain ectopic and orthotopic xenografts and to fully characterize these models. The availability of OS PDXs across the many international PDX platforms and their possible use in PDX clinical trials are also described. We recommend the coupling of next-generation sequencing (NGS) data analysis with functional studies in OS PDXs, as well as the setup of OS PDX clinical trials and co-clinical trials, to enhance the predictive power of experimental evidence and to accelerate the clinical translation of effective genome-guided therapies for this aggressive disease.

## 1. Introduction

Osteosarcoma (OS) is a highly aggressive bone tumor. Representing no more than 0.2% of all malignant tumors, it is classified as a rare tumor, but its social impact is relevant because the primary peak of tumor onset is in childhood and adolescence [1,2,3].

Conventional treatments of OS are based on local surgical control of the disease combined with high dose chemotherapy. First-line conventional drugs include methotrexate, doxorubicin, cisplatin (MAP), and possibly ifosfamide (MAPI) or etoposide. This combined treatment can determine a 5-year survival rate of 60–70% for localized OS, but not for metastatic OS, for which survival drops down to 20–30% [4,5,6]. A combination of gemcitabine and docetaxel is used as a second-line treatment for refractory or relapsed disease, but the survival of such high-risk patients is still dismal. Further attempts to increase survival by intensifying chemotherapy in international randomized controlled trials were unsatisfactory [4,7], and the treatment of metastatic OS still represents an open challenge for oncologists. 

**Figure 1:**
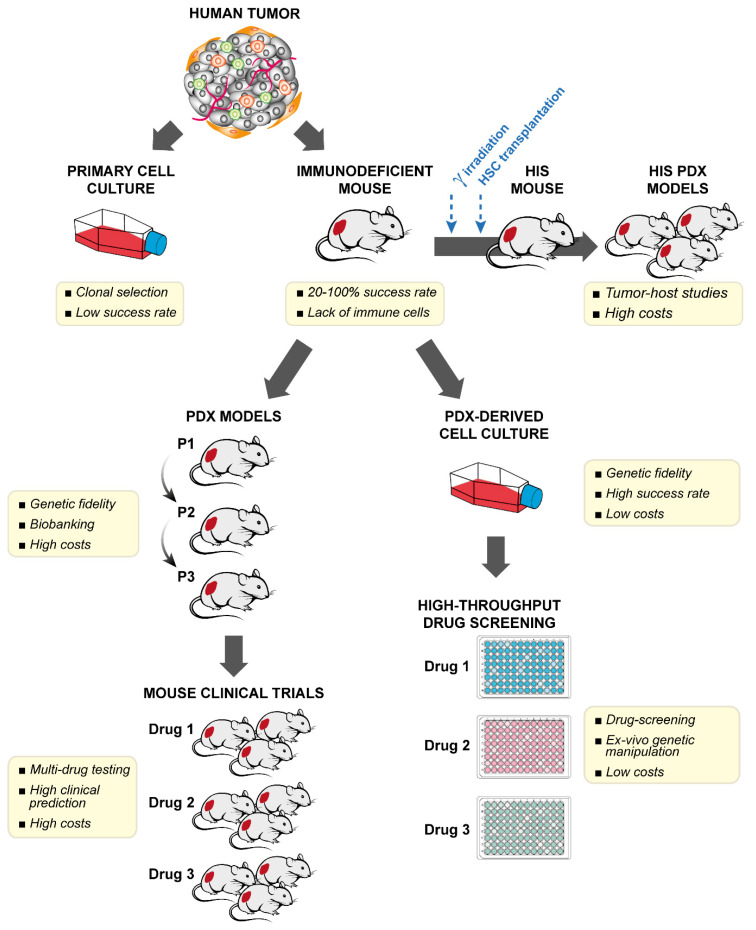
Advantages and limits of the available osteosarcoma patient-derived xenografts (OS PDX) and PDX-derived experimental models. PDX models allow the amplification of patient-derived tumor samples by highly preserving genetic traits and show a higher success rate compared to primary patient-derived cell cultures. In addition, PDX models can be a source of material for the study of tumor-host immune interactions in the human immune system (HIS) reconstituted mice after gamma-irradiation and transplantation of CD34^pos^ human stem cells (HSC). The prompt generation of PDX-derived cell cultures can allow faster and cheaper high throughput drug screening and ex-vivo manipulation compared to PDX models. However, the setup of mouse clinical trials enrolling different OS PDX models can better reproduce the clinical heterogeneity and ensure high predictive value.

Any chance to shape new effective therapeutic strategies can only arise from a deeper understanding of the genetic alterations driving OS and from the clarification of the interactions between OS cells and the complex microenvironment of bone and bone marrow. Recent comprehensive analysis of OS genomic alterations through the application of whole-genome sequencing (WGS) techniques has pointed out how, in 77% of OS cases [8], genomic complexity follows chromothripsis, a catastrophic mutational process during which thousands of clustered chromosomal rearrangements happen in a single event [8,9,10]. Through the simultaneous fragmentation of distinct chromosomal regions and the subsequent imperfect reassembly, chromothripsis is thought to be largely responsible for the chaotic and extensive chromosomal rearrangements that characterize OS, which include multiple somatic copy number alterations and structural changes leading to the simultaneous amplification of oncogenes, loss of tumor suppressors and inactivation of DNA-repair genes [11]. This phenomenon is a notable exception to the conventional wisdom that cancer is caused by the gradual acquisition of genomic rearrangements and somatic mutations. Furthermore, chromothripsis is thought to be largely responsible for the unsuccessful clinical implementation of targeted drugs in OS. Unlike other tumors, in which specific oncogene addictions and actionable mutations have been identified, the widespread mutational load of OS has hampered the identification of specific, common oncogenic drivers that could be exploited for therapy. In the era of precision medicine, the great genetic heterogeneity of OS prompts a call for the systematic molecular profiling of each patient’s tumor to be matched with a personalized therapy in real-time. Even though there is great promise in this approach, the high degree of intra-tumor variability, the rarity of the disease, and the need to prioritize agents for early phase clinical trials highlight the urgency of additional approaches besides molecular tumor profiling to guide treatment decisions. Over the past years, several groups and consortia have developed patient-derived xenograft (PDX) models from OSs as a means to reproduce the clinical situation of each tumor. OS PDXs and new cell lines derived from PDXs have been shown to closely maintain the phenotypic and genotypic properties of the tumor of origin [12,13,14], including genetic and epigenetic features, inter-and intra-tumor heterogeneity, and drug sensitivity [15]. This Review describes recent advancements in OS PDXs, summarizes the methods for ectopic or orthotopic engraftment in immunodeficient mice, the available models, and the international repositories, and illustrates the utility of PDXs in clinical decision-making, such as the categorization of genetic-driven drug responses, to accelerate the translation of new targeted drugs into the clinics. Figure 1 schematizes the major advantages and disadvantages of OS experimental models developed to prospectively guide OS patients toward genome-driven, individualized therapies. 

## 2. Establishment of OS PDX Models

PDX are mouse models of human tumors obtained by directly grafting fresh surgical tumor fragments in immunodeficient mice. In the case of OS, the sources of tumor tissue can be biopsies, surgical resections of the primary tumor (usually after neoadjuvant chemotherapy), or resections of metastatic lesions. All can be used to establish models that represent different steps of tumor progression [16].

The rates of successful OS PDX engraftment and establishment, usually considered over at least three in vivo passages [12,16,17,18], range from 20% to 100% (Table 1). OS PDX engraftment rates are affected by many variables, some intrinsic to the tumor samples, others related to the experimental conditions. For example, samples deriving from biopsies are usually very small in volume but have a high viable cell content derived from the chemotherapy-naive tumor; in contrast, the material obtained from surgical resections is more abundant but can show massive necrosis if it is obtained after chemotherapy and must be inspected by an experienced pathologist to select viable tissue. Post-therapy samples may have a higher content of resistant and more aggressive cells, thus resulting in higher rates of PDX establishment [12]. 

The time span between primary implantation in mice and tumor development can be very long, from one month to almost one year for OS PDX [12]. Therefore, only rarely the PDX is immediately informative for the patient; more often, a well-characterized OS PDX model will be of help for subsequent patients sharing similar tumor features.

### 2.1. Ectopic vs. Orthotopic Models

In ectopic engraftment, human tumor samples are usually inserted in a subcutaneous pocket created in the flank of anesthetized mice. Another site that offers many advantages for ectopic subcutaneous engraftment is the inter-scapular brown fat pad. Subcutaneous implantation is easy to perform, and monitoring of tumor take and growth is readily done [12]. An alternative ectopic site is the subrenal capsule, even though surgery is more complex [13,19]. Additionally, tumor fragments can be dipped in Matrigel prior to implantation to provide further matrix and growth factors. For an exhaustive description of methods to establish PDX from sarcoma samples, see Surdez et al. [20].

For OS, orthotopic implantation can be achieved through intrafemoral or intratibial injection. The orthotopic setting has been frequently advocated as a way to implant tumors in the bone microenvironment that favors metastatic spread [21,22,23,24,25] and has been used to investigate drugs aimed at preventing metastatic progression. However, in a recent study, Maloney et al. [26] demonstrated that the intratibial injection of the mouse OS cell line K7M2 in syngeneic BALB/c mice resulted in direct seeding to the lungs, therefore represents an experimental, rather than a spontaneous model, of metastasis [26]. In addition, intrafemoral and intratibial OS cell injection generally requires the dissociation of the original tumor fragment into single cells, thus leading to the loss of the three-dimensional architecture of the tumor [18].

### 2.2. Animal Models 

PDX establishment mandates the use of immunodeficient mice; however, researchers can consider different options with different levels of immunodeficiency and the related advantages and disadvantages. 

The nude mouse is the oldest immunodeficient model; an epithelial defect leads to the absence of the thymus; hence precursor T cells are generated in the bone marrow but most die because they cannot mature in the thymus. Hence, T cell-mediated immune responses and antibody production are heavily compromised, but innate immunity and natural killer (NK) cells, or extra-thymic maturation of T cells, can hamper the primary engraftment of human tumor fragments. Thanks to the affordable cost and to their general tolerance to cytotoxic treatments and radiotherapy, nude mice are frequently used for the amplification of previously established PDXs or for extensive drug testing, but their use for direct implantation of patient material is generally discouraged due to lower success rate of PDX establishment in comparison with other immunodeficient mouse models obtained more recently.

Cross-breeding of severe combined immunodeficient (SCID) mice and non-obese diabetic (NOD) mice, combined with the knockout of the common interleukin receptor gamma chain (IL2rg^−/−^), produced the highly immunodeficient mouse line Nod Scid Gamma (NSG), which lack mature T, B, and NK cells and are defective in macrophage activity. The very low residual immunity and a long life span make NSG an excellent host for the implantation and growth of solid human tumors; however, their low tolerance to radiation and cytotoxic drug treatments can hamper the study of therapeutic treatments [27,28]. A higher tolerance to toxicity is offered by mice that combine Il2rg and recombinase-activating gene (Rag)-1 or -2 knockouts (e.g., BALB Rag^−/−^;Il2rg^−/−^ or NOD Rag^−/−^;Il2rg^−/−^), which generally have the same level of immune deficiency as NSG mice [28,29]. 

On the whole, the reported percentages of successful engraftment of solid human tumors are roughly proportional to the degree of immunodeficiency (NSG and Rag^−/−^;Il2rg^−/−^ > NOD/SCID > SCID > *nude*) [28]. However, for OS PDXs, variable results have been reported, and no firm conclusion can be drawn [30] (see Table 1 for details). 

The lack of a fully functional immune system and the fact that human stromal components—such as cancer-associated fibroblasts, endothelial cells, and inflammatory cells—are readily replaced by murine counterparts is a major limitation for the study of tumor-host interactions and for the assessment of immunotherapeutic agents [31,32,33,34,35,36]. The development of humanized mice, harboring a human immune system (HIS mice) and/or human stromal microenvironmental components, is a valuable step forward for studies of human onco-immunology, immunotherapy, and immune checkpoint inhibitors [30]. However, such models are intrinsically complex and cumbersome, and the establishment in a mouse of a matched tumor and immune system from the same patient is still a challenging task (Figure 1); the details are summarized in the recent, comprehensive review by Stripecke et al. [37]. 

Spontaneous canine models of OS are capturing greater attention as an alternative to xenotransplants in immunodeficient rodents. In the last 20 years, comparative oncology has seen a great expansion, and the study of naturally occurring cancers in companion animals now provides a suitable system for the advancement of the understanding and management of human cancer, including sarcomas [38]. The incidence of OS is high in large dog breeds, which therefore make a very attractive model for studying the disease in the appropriate cellular context. The results of dog OS studies are translatable to humans, as it has been shown that canine and human OS share remarkable similarities in tumor biology and behavior, including metastatic ability. From a genetic point of view, mutational profiles are comparable, with alterations in P53, RB1, MYC, MTOR, and PTEN found in both species [39,40]. Proteomic profiles and immunohistochemical markers confirmed the high similarity between the two pathological entities [41,42]. Dogs are immunocompetent and offer a suitable environment for testing novel therapeutic approaches, including immunotherapy [43] and oncolytic virotherapy [44]. For extensive reviews of canine OS, see [45,46]. 

### 2.3. OS PDX Validation

Tumors growing as PDX require histological, molecular, and genetic validation in comparison with the original human tumor sample before they are used for any preclinical study. Amplification/use of early passages is recommended to prevent cross-contamination between different PDXs and the onset of clonal selection or of genetic drift; continuous quality assessment should also be implemented. Authentication through short-tandem repeat profiling can assure identity with the patient [12,18,20,54]. Further molecular analyses include whole genome sequencing, gene expression, and whole exome sequencing, copy number alteration (CNA) analysis, structural variation analysis, and point-mutation analysis. 

A large study collecting data of CNAs from both the primary tumor and its derived PDX of 342 unique PDX models across 24 cancer types, including some bone sarcomas, concluded that, at the cohort level, PDX models are genomically representative of the original human tumors [55]. A longitudinal study of CNA across sequential passages of each PDX model revealed that CNA events are more frequent in the initial (first–fourth) in vivo passages, then decrease in later passages after stabilization of selected preexisting subclones. Similar dynamics and rates of CNA acquisition were reported for new in vitro derived human cancer cell lines [55]. A study analyzing the clonal composition of PDXs in relation to the tumor of origin reported that compared to other pediatric tumors, such as neuroblastoma and rhabdomyosarcoma, OS PDX had the best conservation of the clonal profile [18].

Furthermore, Guilhamon et al. [56], studying the tumor epigenomics in two OS PDX reported a limited (1–6%) methylation shift between the first PDX passage and the parental human tumor, followed by minor changes in later passages, thus suggesting that OS PDX are valuable tools also for epigenetic drug testing. However, CNA or epigenetic alterations can occur during PDX propagation, and they may affect genes involved in therapeutic responses, thus altering the drug sensitivity of the PDX in comparison to the patient. As an example of changes that may occur during PDX propagation, it was shown that the apoptosis-related signature could decrease along passages while the proliferation signature could increase [55], in agreement with the shorter tumor latency and increased growth rate usually observed during PDX passaging [12,18].

In general, on-going monitoring of the common genetic traits between PDXs and human parental tumors is highly recommended to validate the predictive value of each model for individual drug responses.

## 3. OS PDX-Derived Cell Cultures and Cell Lines

PDXs are an important tool for the expansion of patient-derived biopsies and closely resemble the original tumor morphologically and molecularly; however, their use for large high-throughput drug testing is discouraged by the high costs and by the cumbersome handling [16]. Under this respect, the establishment of cell lines from PDXs leads to models more suitable to drug screenings, ex vivo genetic manipulation, and to the development of further xenografts [57,58,59]. 

Several studies have demonstrated that PDX-derived cell lines can be easily established [12,13,14,18,50,53]. Nanni et al. [12] showed a significantly higher success rate in the establishment of OS cell lines from PDXs in comparison to primary OS cell cultures obtained directly from the clinical sample; by analyzing gene expression profiles of matching samples from individual patient’s OS, PDX, and cell culture, the authors demonstrated that the PDX better reflected the molecular features of the original tumor than cell cultures directly derived from the patient’s material [12]. Although the underlying causes are not fully understood, the data suggests that the process of generating in vitro cell cultures leads to alterations of biological/genetic features and/or to the loss of distinct cell populations [60,61,62]. Accordingly, Loh et al. observed major discrepancies in the response to chemotherapy between commercial OS cell lines and PDX-derived cells, with U2OS and SaOS2 clustering separately and showing higher chemosensitivity than short-term PDX-derived cell cultures [14]. More recently, Van Cleve and colleagues also confirmed that PDX-derived cell lines retain the characteristics of the original tumor, including histopathology, cytogenetic complexity, osteoblastic activity, and drug sensitivity [63]. Thus, PDX-derived cell lines are a novel system that can provide more consistent models to investigate the molecular pathogenesis of OS and to test novel therapeutic drugs than commercial cell lines with a long history of in vitro passages.

Like PDXs, also PDX-derived cell lines fail to recapitulate the interactions between OS cells and the immune/stromal system. To overcome this limit, three-dimensional (3D) cell cultures are increasingly popular because they may better represent in vivo tissues than conventional 2D cultures, making it possible to co-culture two or more different cell types and to simulate microenvironmental conditions, such as hypoxia and nutrient gradients (for a review, see [64]). 3D culture technologies have led to the development of organoids, which are tiny, self-organized 3D cultures that can be constructed to replicate much of the complexity of an organ [65,66]. The addition of hematopoietic cells in co-cultures with organoids provides the opportunity to study the interactions between the immune microenvironment and tumor cells [67], to test the efficacy of drugs in a more complex environment, and to develop novel treatment delivery techniques. The organoid models developed so far are mainly oriented towards carcinomas [66]. There is an urgent need to develop specific 3D models that consider the mesenchymal nature of sarcoma cells and the complexity of the bone microenvironment. A recent paper by He et al. [68] defined for the first time a protocol to generate organoids from primary and metastatic OS. These models could be maintained and serially propagated for at least six months and retained the cellular morphology and expression of OS markers while preserving a T cell distribution similar to human OS in patients, thus providing a model to explore how OS cells interact with cells of the bone and bone marrow microenvironment [68]. 

Genetic abnormalities have a crucial role in cancer evolution and progression, but the tumor microenvironment also plays a major role in the pathogenesis through the activation of intracellular signaling pathways that promote growth, survival, and migration of cancer cells, as well as resistance to conventional and targeted therapeutic agents [69]. The bone and bone marrow microenvironment that surrounds OS cells is composed of a variety of cell types, including stromal cells, mesenchymal and hematopoietic stem cells, osteoclasts, osteoblasts, dendritic cells, and vascular endothelial cells. There are multiple signal networks between OS cells and those cellular components, which can both enhance and suppress the proliferation of OS cells, as well as that of normal host cells [70,71]. In this context, 3D systems using OS PDX-derived cell lines faithfully recapitulating the behavior of human tumors represent dynamic models that may help scientists to assess the efficacy of therapeutic treatments in an appropriate cellular context. 

## 4. OS PDX Models of Tumor Progression and Metastasis

OS metastasizes almost exclusively to the lungs. The histologic, genetic, and pathologic features of OS cells that guide them to metastasize to this anatomic site are largely unknown. There are also many unknowns concerning the genetic factors that may predispose OS to metastasis and how OS cells spread to the lungs, either by the hematogenous or the lymphatic route or by direct invasion. Considering that almost one-third of the patients with localized disease at diagnosis will develop lung metastases [23,72] and that patients with metastatic OS at diagnosis have a dismal prognosis, there is a strong need to discover anti-metastatic therapies that improve patients’ outcome by inhibiting lung metastasis progression. Recently, some druggable targets uniquely linked to the metastatic phenotype have been identified (see Fan and colleagues for a recent comprehensive review [73]); however, most anti-metastatic therapeutic strategies that derive from this knowledge seem to be only able to hamper the evolution of micrometastases to macroscopic lesions, but not to induce the regression of established disease. Of note, lung metastasis can present in the form of solitary or multiple nodules, and this also impacts surgical management and patient survival since surgical removal of all metastatic foci is essential to improve the outcome [74]. 

In the contest of metastatic dissemination, the major challenge remains to develop adequate preclinical models to assess the anti-metastatic efficacy of targeted drugs.

Many techniques to investigate the metastatic process and to image the steps of metastatic spread have been developed over the years. Some interesting approaches, including chick chorioallantoic membrane models, ex vivo pulmonary metastasis assay, intravital windows for high resolution imaging in the lung for video microscopy of lung metastasis, are excellently reviewed by Fan et al. [73]. All these methods have advantages and disadvantages, including some difficulties in reproducing the priming of metastatic niches by the primary tumor, but they offer novel and complementary information that can accelerate the discovery and characterization of key processes at work during metastatic progression.

Here, we deal with in vivo model systems, with their potentialities and limits, which are summarized as follows:

### 4.1. OS PDX and Metastasis

Obtaining a PDX model able to generate spontaneous metastases is not an easy task. This is true for any kind of tumor, including sarcomas and carcinomas, even when metastatic lesions are used as the starting material. Indeed, most of the OS PDXs do not reproduce the metastatic phenotype in vivo. Some of the causes may be intrinsic features of the tumor; others can be ascribed to the fact that xenografts fail to restore the proper tumor microenvironment and essential tumor-host interactions. A further obstacle comes from the fast local growth PDXs, as animals must be sacrificed because of local tumor size prior to the completion of the metastatic process. Orthotopic implantation generally yields a higher metastatic capacity than subcutaneous implantation, but on the whole, the incidence of metastasis is still much lower in mice than in patients [14,22,75]. 

### 4.2. Genetically-Engineered Models of OS Metastasis

The development of genetically engineered mice (GEMs) [76] has broadened the study of the metastatic process in OS, and may represent a concrete possibility to study spontaneous metastases. A range of experimental approaches has been used to develop GEM models of OS [77]. Many murine OS models have been established to recapitulate P53 and RB mutations of hereditary and sporadic human OS. Such engineered mouse models reproduce many features of human OS, including similar transcriptional signatures and cytogenetic alterations. In a recent elegant study, Moriarity et al. [78] performed a Sleeping Beauty (SB) transposon-based forward genetic screen in mice with and without somatic loss of P53 and identified candidate metastasis driver genes that may be exploited therapeutically. However, it should be noted that the anatomic sites of primary tumor formation in Cre-loxP mice are different than in patients; only in one study [79], tumors arose primarily in long bones. The frequency of distant metastases was low and, with the exception of a P53 knockdown model [79], mostly involved extrapulmonary anatomic locations, unlike human metastases. On the whole, GEM models provide important information for the genetics of OS, but they are overly artificial and simplified model systems as compared to the complex genomic landscape of naturally occurring, metastasizing OS [73].

### 4.3. Large Dog OS and Metastatic Disease

Dog OSs are also gaining attention in the study of the metastatic process. Several studies of comparative oncology have highlighted the genetical, biological, and clinical similarities between metastatic human and canine disease [40,80,81,82], recognizing the value of dog OS as a reliable model to study novel therapeutic agents against metastatic progression [23]. For example, recent positive results in the control of metastatic progression were obtained in a first-in-dog clinical trial combining radiation therapy and immunotherapy with adoptive transfer of autologous natural killer cells expanded ex vivo [83]. A pilot trial in pet dogs with OS was also conducted for the preclinical testing of a new genetically modified vaccine based on highly attenuated *Listeria* expressing immunodominant epitopes of the human HER-2 oncogene/antigen [43]. Vaccination after surgery and chemotherapy, in a setting of minimal residual disease, elicited effective immune responses in dogs and significantly inhibited the development of metastases, thus improving disease-free and overall survival [43].

## 5. OS PDX in International PDX Platforms

An essential issue in research based on the use of PDXs is the availability of high-standard PDX models together with databases that collect their molecular signatures. Several international PDX repositories have been set up in recent years, and guidelines describing the minimal information for the standardization of PDXs (PDX-MI) [84] or humanized mice (MISHUM) [37] were set forth. In the USA, the National Cancer Institute (NCI) established the Pediatric Preclinical Testing Consortium (PPTC, http://www.ncipptc.org (accessed on 21 December 2020)), to obtain xenograft models of pediatric tumors [29]. The NCI was also the founder of the NCI’s PDXNet (https://www.pdxnetwork.org/, accessed on 21 December 2020), which collects at the moment 207 PDX models of 17 different tumor types with the purpose of expanding and standardize the practice of PDX clinical trials. Similarly, the St. Jude Children’s Research Hospital founded the Childhood Solid Tumor Network (CSTN) (https://www.stjude.org/research/resources-data/childhood-solid-tumor-network/, accessed on 21 December 2020) [85], which presently includes samples and genetic profiles of at least 169 PDX models, comprising 35 OS PDXs. 

In 2013, several European and US Institutions started the EurOPDX consortium (https://www.europdx.eu/, accessed on 21 December 2020), collecting more than 1500 PDXs of different tumors, including pediatric tumors and sarcomas. In addition, several European Institutions and companies are involved in a project aimed at the generation of a cohort of at least 400 PDX models of pediatric solid tumors, which will be fully characterized to build a comprehensive platform for drug testing (ITCC-P4 project, https://www.itccp4.eu/, accessed on 21 December 2020). One of the aims of this project is to generate an exhaustive molecular characterization of these models and the matched human tumors. These comprehensive analyses will include low coverage whole genome sequencing (lcWGS), whole exome sequencing (WES), DNA methylation profiling, and RNA sequencing of the PDXs and matched tumors, as well as lcWGS and WES of patient’s germline DNA. 

Additional OS PDXs can be found in other platforms, involving many research companies outsourcing PDX models (Table 2). To help researchers in finding suitable PDX models for their studies or to increase the visibility of their models, the Jackson Laboratory and the European Molecular Biology Laboratory-European Bioinformatics Institute (EMBL-EBI) are co-developing the PDX Finder portal (http://www.pdxfinder.org (accessed on 21 December 2020), a comprehensive open global catalog of PDX models providing links to the many participating repositories, to facilitate and increase PDX-based research collaborations [86]. 

## 6. Innovative Therapies and Genome-Driven Approaches Evaluated in OS PDX

An update of current innovative therapeutic strategies against OS can be found in recent reviews [7,87]. Many targeted agents demonstrating anti-tumor responses in preclinical or early-phase clinical studies failed to reach the thresholds of efficacy to justify their inclusion in larger clinical trials. Such disappointing results support the need to improve the quality of preclinical models in the screening of novel agents and define effective schedules. In 2013 the PPTC of the NCI demonstrated that OS PDXs provide an efficient platform to test the anti-tumor effects of new agents [88], and several studies have shown the efficacy of various targeted drugs against OS PDXs or PDX-derived cell line models [14,89,90,91]. However, such studies mostly ignored the genomic features of the experimental models. Given the genetic heterogeneity of OS, this lack of information very likely represents a major limitation for the successful clinical translation of experimental results. To reduce discrepancies and errors in molecular studies and drug screening tests, several researchers undertook a comprehensive genetic characterization of xenografts and original tumors and tested agents according to the genetic results [92]. For example, in the study of Sayles and co-workers [13], a large panel of OS PDX carrying different genomic alterations was used to test the efficacy of “genome-matched” targeted therapies. Genetic analyses allowed the authors to recognize six targetable driver pathways: MYC gain, CDK4/FOXM1 gain, PTEN loss/AKT gain, AURKB gain, VEGFA gain, and CCNE1 gain. The growth of PDXs carrying the selected alteration was significantly inhibited when the matching drug was used; in contrast, the administration of drugs targeting pathways not matching the genetic profile of the OS PDXs was found to be completely ineffective. A similar approach was employed by Pandya et al. to prioritize drugs effective against high-risk OS [53]. The authors used as a first step a systems biology approach based on public databases to integrate targetable genetic alterations and dysregulated pathways with patients’ outcomes, followed by the identification of molecularly characterized OS models harboring the specific alterations and by the selection of clinically relevant inhibitors targeting the dysregulated network. Table 3 summarizes candidate targetable pathways, their frequency in cohorts of OS patients, and major results when genome-matched therapies were tested in genome-selected OS PDX models. As predicted, these studies found higher therapeutic efficacy when therapies were selected according to the genetic traits of the OS PDXs, supporting the need to integrate omic genetic analysis for each patient with suitable PDX models, to prioritize actionable therapeutic targets and validate drug responses [13].

## 7. Mouse PDX Clinical Trials and Co-Clinical Trials

Following the ideas outlined above, mouse clinical trials (MCTs) and co-clinical trials, which are MCTs run in parallel and in real-time with human clinical trials, were recently started. MCTs are preferentially based on the subcutaneous engraftment of PDX tumors, a procedure that allows a rapid setup and easy detection of tumor growth, and include PDX models established from the patients that are enrolled in a clinical trial [95,96]. PDXs used in a co-clinical trial are indeed also called “avatars” to indicate that these models can function as a “mirror” of the donor patient, with the main aim to assess patient drug responses. These experimental trials demonstrated accurate clinical prediction in multiple studies, [97,98], and can predict both the development of resistance to first-line therapy and the response to second-line therapy before these events happen in the donor patient [99]. 

MCT and co-clinical trials offer several advantages, particularly for a rare and complex tumor-like OS. Unlike human patients, a PDX model can be treated in parallel with the same drug used for the patient and with different drugs, or with novel combinations. While in a clinical trial, a patient is only enrolled in one arm, in MCTs, different mice bearing the same PDX are available and can be enrolled in different arms of the same trial. The number of mice can be easily increased to reach the desired statistical power and to evaluate potential side effects. Tumor volume can be measured frequently, and errors can be quantified as multiple mice are used in each arm. Moreover, in genome-driven drug testing, it is possible to enroll enough PDX models with or without the required genetic profile to ensure statistical significance.

As in clinical trials, MCT setup requires the evaluation of statistical power and sample size calculations to determine the required number of different PDX models and the number of mice for each PDX model [100]. In both drug and vehicle groups, a balanced n:n design, with *n* (≥1) mice per PDX, per group, must be considered. The number of different PDXs needed to achieve sufficient statistic power can change as a function of the efficacy of the tested drug. In general, a higher number of mice for each PDX can ensure a higher statistical power for the evaluation of drug efficacy against each tumor entity; however, to better account for clinical heterogeneity, an experimental design using a larger number of PDX models, with fewer mice or even one mouse per PDX per group, could be preferred [100].

Among the trials listed in https://clinicaltrials.gov (accessed on 21 December 2020), the use of OS PDX was proposed by a trial entitled “Patient-derived Xenograft (PDX) Modeling to Test Drug Response for High-grade Osteosarcoma” presented by Guo Wei, Peking (NCT 03358628). The purpose of the study (not yet recruiting) is to use clinical specimens from OS patients to establish and genomically profile OS PDX in mice. Matched treatment recommendations based on genomic profiling and in vivo PDX drug testing results will be made available, potentially providing patients with individualized cancer treatment options. In the phase 2 clinical study (NCT04417062) proposed by the Dana-Farber Cancer Institute to evaluate the effectiveness of using two drugs (olaparib and ceralasertib) to treat patients with OS that have not responded to conventional treatments, the derivation of PDX models and paired pre/post-treatment tumor samples are included and may offer additional treatment opportunities [101,102].

Most recent clinical trials are adapting their structure to take into account the increasing landscape of multi-omic data and are developing dynamic studies able to cope not only with tumor heterogeneity but also with tumor evolution (metastatic progression and drug resistance) under the selective pressure exerted by therapeutic treatments [103]. 

In general, the possibility to perform mouse PDX clinical trials and co-clinical trials will establish an invaluable translational oncology platform to implement precision-guided therapy even in rare tumors.

## 8. Critical Issues and Perspectives

- OS is a rare, highly malignant tumor with a high level of inter- and intra-tumor heterogeneity. Rarity and complexity severely hampered the development of innovative treatments, and very few targeted agents have achieved clinical endpoints for OS.
- OS lack pharmacologically tractable DNA alterations. Thus, the application of precision medicine requires a deeper understanding of cancer biology. There is a need to explore oncogenic mechanisms beyond the identification of genomic driver aberrations and to incorporate new methodologies, such as transcriptional analysis and the development of suitable experimental models.
- PDX models are an important tool for the expansion of patient-derived biopsies. This is highly relevant for rare tumors that are frequently diagnosed by needle-biopsy, such as OS. However, while they closely resemble the original tumor specimen at the morphological and molecular level, they might be overly expensive and cumbersome for many laboratories. In addition, very few laboratories may have direct access to patient material in real-time. Multicenter collaborative networks are highly recommended to increase the number of OS models and to analyze data following standardized procedures.
- PDXs may be unsuitable for large high-throughput drug testing, whereas PDX-derived cell cultures can be easily established and faithfully represent the original tumor. They are a valuable platform for drug response profiling. PDX-derived cell lines are readily exchangeable among laboratories, which may help the harmonization of data, even for rare tumors.
- Therapeutic failure for patients with OS still involves the development of metastasis to the lungs, despite effective and complete control of the primary tumor. The complexity of the metastatic cascade is difficult to be modeled in vitro. 3D cultures could represent an excellent option to mimic the interactions of tumor cells within the tumor microenvironment. 3D printing is being used to create bone scaffolds that can incorporate a variety of cells, growth factors, and drugs (for a review, see [104]). Even if some technical issues are to be solved and extensive optimization is still needed, these scaffolds have the potential to accelerate the transition from the laboratory to patient care. Thus, their development is highly recommended.

## 9. Concluding Remarks

**Figure 2:**
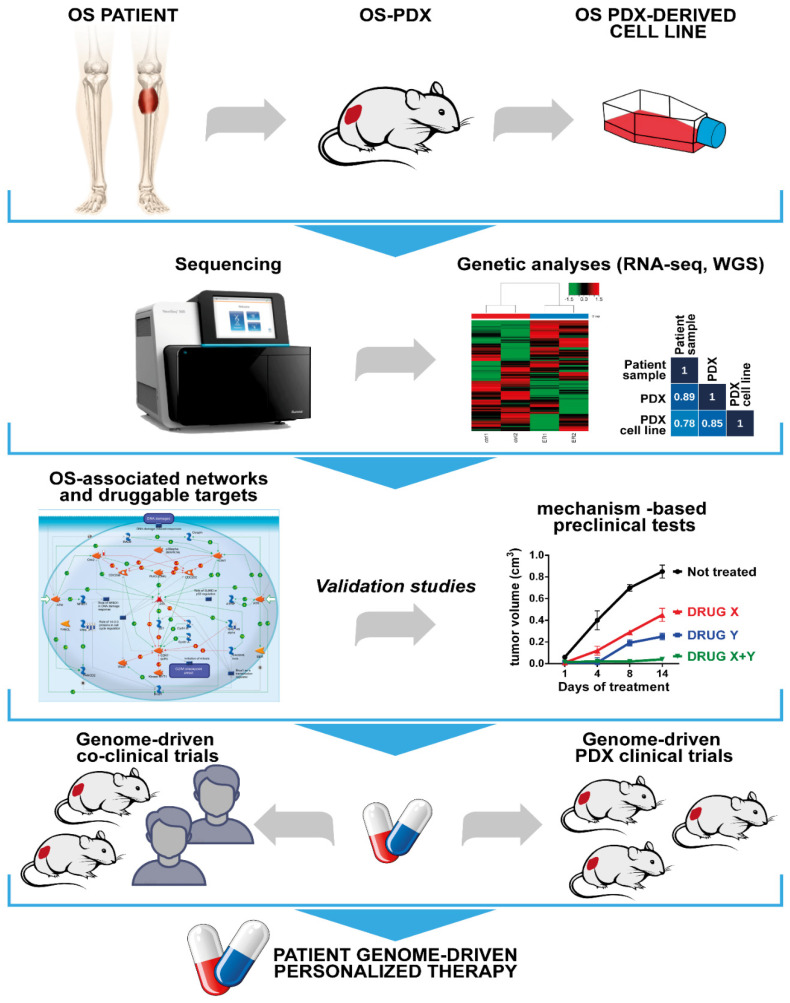
Development of genome-driven therapeutic approaches for OS: From PDX and preclinical in vitro and in vivo studies to molecular data and DNA/RNA-guided personalized medicine. Patient tumors, PDXs, and PDX-derived cell lines should undergo comprehensive molecular characterization, including sequencing and genetic analyses. Targeted therapies should be selected based on genomic analysis and assessed in mechanism-based preclinical studies. The results obtained can potentially guide the design of genome-driven, personalized approaches based on PDX models, including co-clinical trials and PDX clinical trials.

OS therapy has remained unchanged for a long time, but the recent application of multi-omic approaches made possible the evaluation of the complex and heterogeneous genetic alterations that characterize each OS. Therapeutic choices made on the basis of actionable targets identified by means of DNA sequencing have been implemented in several tumors [105,106]. However, systematic analysis of DNA-actionable mutations is not sufficient to guide the design of personalized treatments in OS. RNA-guided therapies should be selected when there is no actionable DNA alteration or when the DNA-selected drugs are unavailable. In addition, the extreme genetic intra- and inter-tumor heterogeneity that characterize OS, the rarity of the disease, and the frequent failures of phase I and phase II clinical trials attempted so far have prompted scientists to accept the idea that any new kind of clinical trial should be based on NGS data analysis coupled with functional studies of models that can reproduce the dynamic evolution of each individual cancer. PDXs and PDX-derived cell lines are an important platform for drug screenings. They mirror the genetic alterations of original tumors, particularly of high-risk and chemotherapy-resistant diseases, and may be useful for drug development prioritization. Since not all human OSs are able to give rise or have the chance to generate a PDX model, the use of representative, genetically well-characterized OS PDXs will produce benefits, in terms of genome-matched therapy, for OS patients sharing a similar genetic profile. The availability of large and shared repositories of PDX and PDX-derived cell lines, coupled with an extensively annotated genomic dataset of somatic oncogenic alterations of the original tumors, should be favored with the aim to offer drug response profiling as a tool for therapeutic decisions for individual patients in the clinics. Figure 2 summarizes the steps that are recommended to prospectively navigate OS patients towards therapeutic approaches guided by the principles described in this Review.

## Figures and Tables

**Table 1:** OS PDX establishment and OS PDX-derived cell lines in most relevant collections.

Studies	Immunodeficient Mouse Model	First Tumor Engraftment Site	Rate of Engraftment (PDX/Implanted Tumors)	PDX Validation and Molecular Annotations	OS PDX-Derived Cell Cultures
Ishii 1983 [47]	BALB/c Nude	Sc	80% (24/30)	Histology	No
Bauer 1986 [48]	BALB/c Nude	Sc	6 OS PDX	Histology, ploidy, Ki67	No
Meyer 1990 [49]	CBA/CaJ (Thymectomy and irradiation)	Sc	24% (8/33)	Histology, ploidy, LDH	No
Fujisaki 1995 [50]	Nude	Sc	62% (21/34)	NA	PDX-derived short-term cell cultures for in vitro evaluation of drug sensitivity
Bruheim 2004 [17]	BALB/c Nude	Sc	20% (11/55)	Histology	No
Monsma 2012 [51]	Nude	Sc	100% (3/3)	Histology, Genomic, gene expression	No
Kresse 2012 [52]	BALB/c Nude	Sc	9 OS PDX	Genomic	No
Stewart 2017 [18]	NSG	Orthotopic (intrafemural) in matrigel	49% (15/31)	Histology, SATB2, Genomic	PDX-derived short-term cell cultures for in vitro evaluation of drug sensitivity
Sayles 2019 [13]	NSG	Subrenal capsule in matrigel	50% (15/30)	Histology, Genomic	PDX-derived cell cultures (WGS validated) for in vitro evaluation of drug sensitivity
Nanni 2019 [12]	NSG, BALB Rag2^−/−^,Il2rg^−/−^	Sc (interscapular fat pad)	36% (22/61)	Histology, SATB2, gene expression	Several Patient and PDX-derived cell cultures
Loh 2019 [14]	Nude CD1NSG	Sc and then orthotopic (intrafemural) in matrigel	8 OS PDX	Genomic	PDX-derived short-term cell cultures for in vitro evaluation of drug sensitivity
Pandya 2020 [53]	NSG	Sc	1 OS PDX	STR analysis, Genomic	PDX-derived cell culture (WGS validated) for in vitro evaluation of drug sensitivity

Sc, subcutaneous; NA, not available; STR, short-tandem repeat; WGS, whole genome sequencing.

**Table 2:** List of the most relevant international PDX repositories including OS models.

PDX Platforms	Available PDXs	Internet Link
Pediatric Preclinical Testing Consortium (PPTC), National Cancer Institute (NCI), USA [29]	PDXs of pediatric tumors	http://www.ncipptc.org *
PDXNet, National Cancer Institute (NCI), USA	207 PDX models of 17 different tumor types, including sarcomas	https://www.pdxnetwork.org/ *
Childhood Solid Tumor Network (CSTN), St. Jude Children’s Research Hospital, USA [85]	169 PDX models, including 35 OS PDXs	https://www.stjude.org/research/resources-data/childhood-solid-tumor-network.html *
EurOPDX consortium, many European Universities	1500 PDXs including sarcomas	https://www.europdx.eu/ *
ITCC-P4, many European Institutions and companies	400 PDXs of pediatric solid tumors including OS PDX	https://www.itccp4.eu/ *
CrownBio	2500 PDXs including 15 OS PDXs	https://www.crownbio.com/ *
Champions Oncology	1000 PDXs, including 150 models of adult and pediatric sarcomas	https://championsoncology.com/ *
The Jackson Laboratory’s PDX Resource	more than 400 PDX models, including six OS PDXs	https://www.jax.org/ *
DNA Link	300 PDX models	http://www.pdx.dnalink.com/index *
PDXfinder, The Jackson Laboratory and the European Bioinformatics Institute of the European Molecular Biological Laboratory (EMBL-EBI) [86]	4372 different models, with 84 OS PDXs	http://www.pdxfinder.org *

* Accessed on 21 December 2020.

**Table 3:** Genome-driven targeted therapies based on matching evaluation of genomic alteration in OS patients and OS PDXs.

Targetable Pathway	Rate of Alteration in OS Patient Cohorts	Targeted Drugs	OS PDX with the Alteration	Responses in Genome-Matched OS PDX (Range of % Tumor Growth Inhibition)
MYC (8q24.21 gain)		CDK inhibitor AT7519	Two OS PDXs with >12 CN (OS152 and OS186)	86–97% [13] *
	8–39% [13,93]	BRD4 inhibitor JQ1	Two OS PDXs with >12 CN (OS152 and OS186)	No effect on tumor growth in both models [13] *
		Combination of BETi/OTX-015 and CHK1i/SRA737	One OS PDX with four CN (TT2-77 PDX)	Around 90% [53] ^#^
CDK4 (12q14.1 gain)	11–14% [13,93]	Palbociclib (CDK4/6 inhibitor)	Three OS PDXs (OS156, OS128, and OS107)	61–111% [13] *
AURKB (17p13.1 gain)	6–13% [13,93]	AZD1152	One OS PDX (OS107)	57% [13] *
VEGFA (6p12–21 gain)	22–24% [13,93,94]	Sorafenib	One OS PDX (OS106)	79% [13] *
Cyclin E (CCNE1) (19q12 gain)	8–33% [13,93]	Dinaciclib (SCH 727965) (inhibitor of CDK1,2,5,9)	Three OS PDXs (OS457, OS106, and OS452)	54–94% [13] *
PTEN (10q23.21 loss)	4–56% [13,93]	MK2206	One OS PDX (OS052, PTEN loss) One OS PDX (OS525, AKT gain)	61–67% [13] *

* The study was performed using PDX-derived cells injected subcutaneously into the flank of Nod Scid Gamma (NSG) mice; ^#^ The study was performed using a PDX model subcutaneously implanted into the flank of NSG mice.
